# Schistosome Infection and Schistosome-Derived Products as Modulators for the Prevention and Alleviation of Immunological Disorders

**DOI:** 10.3389/fimmu.2021.619776

**Published:** 2021-02-22

**Authors:** Yi Mu, Donald P. McManus, Nan Hou, Pengfei Cai

**Affiliations:** ^1^Molecular Parasitology Laboratory, Infectious Diseases Program, QIMR Berghofer Medical Research Institute, Brisbane, QLD, Australia; ^2^NHC Key Laboratory of Systems Biology of Pathogens, Institute of Pathogen Biology, Chinese Academy of Medical Sciences & Peking Union Medical College, Beijing, China

**Keywords:** schistosome, autoimmune and inflammatory diseases, allergic asthma, colitis, diabetes, sepsis, cystitis, cancer

## Abstract

Parasitic helminths, comprising the flatworms (tapeworms and flukes) and nematodes (roundworms), have plagued humans persistently over a considerable period of time. It is now known that the degree of exposure to these and other pathogens inversely correlates with the incidence of both T helper 1 (Th1)-mediated autoimmunity and Th2-mediated allergy. Accordingly, there has been recent increased interest in utilizing active helminth worm infections and helminth-derived products for the treatment of human autoimmune and inflammatory diseases and to alleviate disease severity. Indeed, there is an accumulating list of novel helminth derived molecules, including proteins, peptides, and microRNAs, that have been shown to exhibit therapeutic potential in a variety of disease models. Here we consider the blood-dwelling schistosome flukes, which have evolved subtle immune regulatory mechanisms that promote parasite survival but at the same time minimize host tissue immunopathology. We review and discuss the recent advances in using schistosome infection and schistosome-derived products as therapeutics to treat or mitigate human immune-related disorders, including allergic asthma, arthritis, colitis, diabetes, sepsis, cystitis, and cancer.

## Introduction

*Schistosoma* spp. are digenetic trematodes that cause schistosomiasis (Bilharzia), a disease afflicting over 230 million individuals in developing countries in Africa, South America, and Asia (1). Schistosomiasis was reported as being responsible for an estimated global burden of 1.4 million disability adjusted life years (DALYs) in 2017 (2). Three schistosome species, *Schistosoma haematobium*, *S. mansoni*, and *S. japonicum* are the most clinically relevant. Liver fibrosis is the main contributor responsible for the morbidity and mortality among individuals with chronic hepatosplenic schistosomiasis. Currently, there is no practical vaccine available for schistosomiasis. Control of the disease relies predominantly on mass drug administration (MDA) programs incorporating the drug praziquantel (3).

Schistosomes are dioecious and have a lifecycle involving an aquatic snail as an intermediate host and a mammalian definitive host (4). During the schistosome lifecycle, free-swimming cercariae penetrate a mammalian host. After skin penetration, the larvae transform into schistosomula, which can reach the heart and lungs within 3–5 days, and within 2 weeks the juvenile worms migrate to the hepatic portal system, where they pair up and become sexually mature. The paired schistosome adult worms then migrate to the pelvic venous plexus (*S. haematobium*) or the mesenteric veins (*S. mansoni* and *S. japonicum*), where the female worms lay eggs intravascularly, with patency times varying among species. Eggs disseminate through the blood flow and many are entrapped in host tissues, such as the liver and intestine, driving the host immune inflammatory response, which in turn help discharge the remaining eggs from the definitive mammalian host. After release from the definitive host, the eggs hatch in freshwater and transform into miracidia, which penetrate a specific snail intermediate host, developing asexually into mother and then daughter sporocysts; these produce cercariae that are released into water to continue the life cycle (5). Adult schistosomes can dwell in the blood vessels of the definitive hosts for decades, despite being continually exposed to this immunologically harsh microenvironment.

The long period of host/parasite co-evolution has resulted in schistosomes modulating the host immune response during infection using intricate molecular mechanisms. The host immune response elicited due to schistosome infection is polarized as it progresses, going from i) an initial T helper type 1 (Th1) response against migrating immature and mature parasites involving IL-12, interferon-γ (IFN-γ) and tumor necrosis factor-α (TNF-α), ii) a switch to a powerful Th2 response, which is primarily induced by egg antigens, with an elevation in the Th2-type cytokines interleukin (IL)-4, IL-5, IL-9, and IL-13, under the control of regulatory T-cells (6, 7), and finally iii) a chronic regulatory phase with a reduced but still predominant Th2 response due to a prolonged regulatory T-cell environment involving IL-10 and TGF-β (6, 8, 9). Work with gene-deficient mice has shown that during the acute phase of a schistosome infection, the inability to drive Th2-type and anti-inflammatory responses is associated with a severe condition characterized by cachexia and significant host mortality (9–11). A critical event in the schistosome life cycle is the excretion of eggs from the mammalian host to the external environment, a process that requires the host evoking CD4^+^ T-cell-induced granulomatous inflammation to facilitate the passage of eggs through the intestine or bladder (12). However, excessive polarization of the Th2 immune response at the chronic stage can lead to potentially life-threatening pathology characterized by periportal fibrosis and portal hypertension (13); this in turn triggers the host to produce intrinsic factors that elicit a more balanced regulatory anti-fibrotic immune response to restrict the deleterious effects of infection (14).

In areas highly endemic for human schistosomiasis, schistosome re-infection is a frequent event. Naturally infected individuals can develop a state of concomitant immunity to control worm numbers in the host by killing newly invading larvae with a modified Th2 pulmonary response (8, 15). Although the exact mechanism(s) underpinning concomitant immunity remain unclear, it has been suggested that a rich source of potential immunomodulatory molecules secreted by previously established adult worms and/or schistosomula surface antigens could interact with and stimulate the host to develop an immune response targeting larval worms (16–18). Collectively, these observations imply that the regulatory immune response induced by the worm and/or worm-derived components represents a key component for the mutual benefit of both the host (pathology limitation) and the parasite (survival and proliferation). Indeed, observations in murine models and in humans show that regulatory subsets of CD4^+^ T cells (Tregs), both naïve and induced Tregs, play an important role in balancing the Th1/Th2 response and in modulation of schistosomiasis-induced immunopathology leading to granuloma formation and fibrosis both in the liver and intestine (19–22).

## Therapeutic Potential of Live Schistosome Infection and Schistosome Products for Immunological Diseases

Pivotal research published in the late 1980s by David Strachan, an epidemiologist at the London School of Hygiene and Tropical Medicine, showed in an investigation involving more than 17,000 children that those in larger households had fewer instances of hay fever (23). Subsequent research suggested that children living in very clean environments appeared to increase an individual’s susceptibility to a range of other conditions. This is what is so-called the hygiene hypothesis which has been supported by epidemiological evidence and experimental studies and was extended to other allergic diseases and subsequently to autoimmune diseases (24). However, the term ‘hygiene hypothesis’ is vague and misleading, and has been widely criticized (25). Accordingly, the concept was later reformulated as the ‘Old Friends’ hypothesis which argues that early exposure to the vital microbes was not due to measles, colds or other childhood (crowd) infections, but rather microbes already present during primate evolution and in hunter-gatherer times when the human immune system was in the process of being shaped (26). With this concept in mind, parasitic helminths that reside in a chronic state in humans, and are tolerated by the immune system, can be regarded as “Old Friends”.

**Figure 1:**
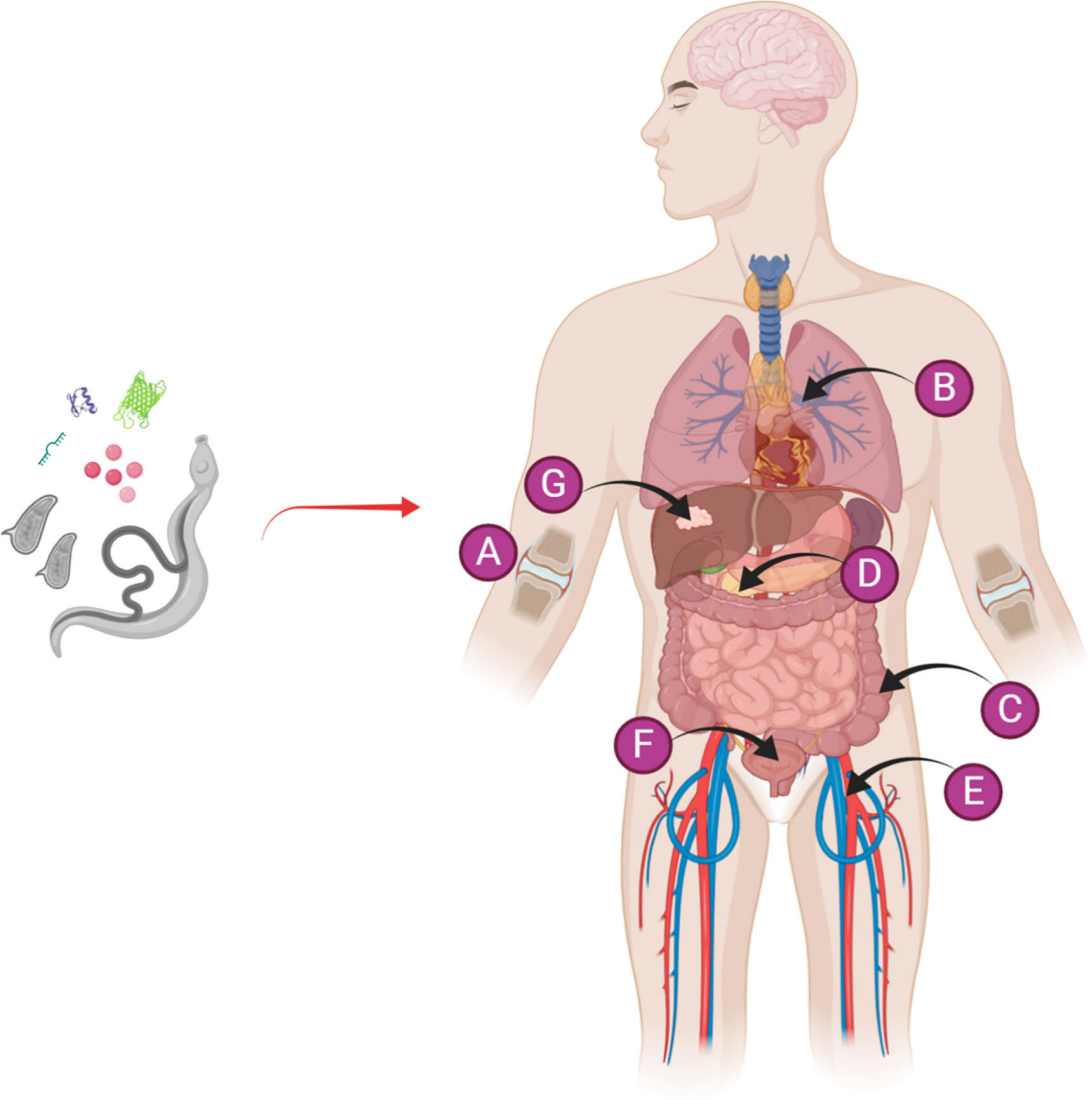
Diagrammatic representation of the use of a live schistosome infection or schistosome-derived products for the prevention/alleviation of a variety of autoimmune and inflammatory diseases, including **(A)** arthritis, **(B)** allergic asthma, **(C)** colitis, **(D)** diabetes, **(E)** sepsis, **(F)** cystitis, and **(G)** cancer. **Figure 1** was created with Biorender.com.

The proclivity for schistosomes to orchestrate immunomodulatory effects on the host immune system combined with the concept of the hygiene hypothesis form the basis for developing therapeutics to protect individuals against the onset of various forms of autoimmune and inflammatory diseases including arthritis, allergic asthma, diabetes, colitis, sepsis and cancer, or to ameliorate their severity (**Figure 1**). In this context, Osada & Kanazawa (27) reviewed the modulatory effects of a concurrent schistosome infection, and the injection of whole eggs, soluble egg antigens (SEA), a soluble antigen preparation of adult schistosomes (SWAP), or recombinant parasite proteins on immunological disorders. Due to the potential safety issues and the accompanying side effects in humans that might be evoked by a live worm infection or the injection of eggs, the use of SEA, SWAP and schistosome secreted and surface-exposed molecules as modulators of the immune system was revisited by Janssen et al. (28). SEA is a complex mixture of phosphate buffered saline-soluble (mainly secreted and cytoplasmic) molecules obtained from mechanically disrupted eggs. Similar to schistosome eggs, SEA is highly antigenic and can induce the activation of a considerable immune response. As with a live schistosome infection, SEA can activate and modulate both the innate immune response through interacting with dendritic cells (DC), macrophages, natural killer T (NKT) cells, eosinophils and basophils, and the adaptive immune system by acting on T cells; this results in the upregulation of anti-inflammatory cytokines and the down-regulation of pro-inflammatory cytokines (29, 30). Individual *Schistosoma*-derived components including recombinant proteins, such as rSjcystatin and rSmKI-1, peptides, and small RNAs, have been recently explored as potential therapeutic targets (**Table 1**). It has been argued that intact helminth parasites may be superior to helminth-derived products for treating chronic inflammatory-associated diseases in humans (59), but this needs to be validated in the case of schistosomes.

**Table 1:** Recent studies of schistosome infection and schistosome-derived products shown to suppress immunological disorders.

Disease	Animal model/cell	Species	Treatment	Association/Modulatory effect	Reference
Rheumatoid arthritis	CIA mouse model	*S. mansoni*	Cercarial infection prior to modelling	Splenic IL-17A↓, TNF-α↓, IFN-γ↓, IL-4↑, IL-10↑	(31)
	CFA-induced AA rat model	*S. mansoni*	Intradermal (ID) injection of ASMA after modelling	IL-17↓, IL-10↑, IFN-γ↑ in serum; Tregs↑ in joint tissues	(32)
	CIA mouse model	*S. japonicum*	Intraperitoneal (IP) injection of rSjCystatin prior to CIA modelling	Splenic IL-4↑, IL-10↑, IFN-γ↓, IL-6↓, IL-17↓, TNF-α↓; collagen specific IgG1↑, IgG2a↓	(33)
	CIA mouse model	*S. japonicum*	Subcutaneous (SC) injection of SJMHE1 prior to and after modelling	Splenic IFN-γ↓, IL-22↓, TNF-α↓, IL-6↓, IL-17↓, IL-10↑, *TGF-β1*↑, Tregs↑	(34)
	MSU-induced gout arthritis model	*S. mansoni*	Intravenous (IV) injection of SmKI-1 after modelling	IL-1β↓ in periarticular knee tissue; neutrophil migration↓ into pleural cavity	(35)
Allergy and asthma	OVA/alum-induced AAI	*S. mansoni*	IP injections of eggs prior to sensitization	Eosinophilia↓ in BAL and lungs, OVA-specific Th2 cytokines↓ in lungs, moDCs↓	(36)
	OVA/alum-induced AAI	*S. mansoni*	IP injection of Smteg during sensitization	IL-5↓, IL-13↓, IL-25↓, CCL11↓, anti-OVA IgE↓, IL-10↑ in lungs	(37)
	PMBCs from asthmatic subjects	*S. mansoni*	PMBCs from asthmatic subjects stimulated with Sm29 and Sm29TSP-2 in the presence of *Der p1*	CD4^+^CD25^hi^ T lymphocytes↑, CD4^+^CD25^low^ T lymphocytes↓, IL-10↑ in the supernatants	(38)
	OVA/alum-induced AAI	*S. japonicum*	Injection of SjP40 peptides (1^st^ in the left footpad and base of tail, 2^nd^ IP) before and in the course of OVA sensitization	IFN‐γ↑, IL‐4↓, IL‐5↓, IL‐13↓ in splenocytes; OVA‐specific IgE↓; IL‐4↓ and IFN‐γ↑ in BALF	(39)
	OVA/alum-induced AAI	*S. japonicum*	SC injection of SJMHE1 during sensitization and challenge	IL‐4↓ in splenocytes; IL‐4↓, IL‐5↓ and IL‐17↓ in the lungs; IFN‐γ↑, IL‐10↑ and IL‐35↑ in the lungs	(40)
	HDM-induced AAI	*S. japonicum*	Cercarial infection prior to modelling	IL‐4↓in BALF and the lungs, IL‐17↓ in BALF	(41)
Colitis	DSS-induced colitis mice	*S. japonicum*	Cercarial infection prior to modelling	IL-6↓, IL-2↓, IL-10↓, IL-17a↓, IFN-γ↓ and TNF-α↓ in serum; ER stress markers IRE1α↓ and IRE1β↓ in colon tissue;	(42)
	DSS-induced colitis mice	*S. mansoni*	Male-only cercariae infection prior to modelling	IFN-γ↓, IL-4↑, IL-10↑, and IL-17↑ in MLNs	(43)
	DSS-induced colitis mice	*S. mansoni*	IP injection of freeze/thaw-killed eggs	IFN-γ↓, IL-2↓, IL-4↓, IL-10↑ in serum; Tregs↑ in colon tissue	(44)
	Colitis induced by chronic adoptive T-cell transfer in SCID mice	*S. mansoni*	SEA injection after modelling	Th2 cells↑, Th17 cells↓ in colon tissue	(45)
	DSS-induced colitis mice	*S. japonicum*	IP injection of rSj16 after modelling	TNF-α↓, IFN-γ↓, IL-17a↓, Chil3↓, TGF-β↑, IL-10↑ in colon tissue; Tregs↑ in spleen and MLNs	(46)
	TNBS-induced colitis mice	*S. japonicum*	IP injection of rSjcystatin after modelling	IL-4↑ and IL-13↑ in colon; IFN-γ↓, IL-10↑ and TGF-β↑ in MLNs and spleen	(47)
	TNBS-induced colitis mice	*S. haematobium*	SC injection of P28GST prior to modelling	IFN-γ↓, IL-4↑, IL-13↑ and eosinophil infiltration↑ in colon tissue	(48)
Type 1 diabetes	NOD mice	*S. japonicum*	IP injection of rSjCystatin prior to modelling	IFN‐γ↓, IL-4↑, IL‐10↑, TGF‐β↑, and Tregs↑ in spleen and PLN	(49)
	NOD mice	*S. japonicum*	IP injection of rSjFBPA prior to modelling	IFN‐γ↓, IL-4↑, IL‐10↑, TGF‐β↑, and Tregs↑ in spleen and PLN	(49)
	STZ-induced diabetic mice	*S. mansoni*	Cercarial infection prior to modelling	M2 macrophage markers, Arg-1↑ and Ym1↑ in PLN	(50)
Type 2 diabetes	Diet‐induced obese mice	*S. mansoni*	Cercarial infection/IP injection of SEA after modelling	Th2 cells↑ and eosinophilia↑ in WAT and liver; M2 macrophage↑ in WAT	(51)
	*Lep*r^db/db^ mice	*S. japonicum*	IP injection of SEA	IL-4↑, IL-5↑,Tregs↑ in splenocytes	(52)
Sepsis	LPS-induced septic mice	*S. japonicum*	Cercarial infection prior to modelling	M2 macrophage↑, M1 macrophage↓ in peritoneal lavage cells	(53)
	LPS-induced septic mice	*S. japonicum*	IP injection of rSj-Cys after modelling	IL-10↑, TGF-β1↑,TNF-α↓, IL-6↓, IL-1β↓ in serum; MyD88↓ in liver, kidney and lungs	(54)
	LPS-induced septic mice	*S. japonicum*	IP injection of rSj-Cys after modelling	IL-6↓, TNF-α↓, IL-10↑ in serum	(55)
Cystitis	Ifosfamide-induced hemorrhagic cystitis model	*S. haematobium*	IV injection of IPSE prior to modelling	IL-1β-TNFα-IL-6 pathways↓, interferon signaling↓, oxidative stress↓ in bladder	(56)
Cancer	Mouse model of DMH-induced colon cancer	*S. mansoni*	IP injection of ASMA after modelling	IL-17↓, IL-10↑ in serum; splenic CD4^+^T-cells↑, intestinal FoxP3^+^ Tregs↑	(57)
	Hepatoma cell lines/mice bearing Hepa1-6 xenografts	*S. japonicum*	Transfection of Sja-miR-3096 mimics into cell lines/IV injection of Sja-miR-3096 mimics into mice bearing Hepa1-6 xenografts	*PIK3C2A*↓; tumor weight↓	(58)

AAI, airway allergic inflammation; ASMA, autoclaved antigen derived from S. mansoni cercariae; CFA-induced AA, complete Freund’s adjuvant-induced adjuvant arthritis; CHC, chemotherapy-induced hemorrhagic cystitis; CIA, collagen-induced arthritis; DMH, 1,2-dimethylhydrazine; ID, Intradermal; IP, Intraperitoneal; IV, intravenous; MLNs, mesenteric lymph nodes; MSU, monosodium urate; NOD, non-obese diabetic; SC, subcutaneous; SCID, severe combined immunodeficiency; PLN, pancreatic lymph node; WAT, white adipose tissue.

Given the capacity of schistosomes to hasten or nullify the development of inflammatory and autoimmune diseases, some of the disorders that have shown improved outcomes after schistosome infection or exposure to schistosome components are now discussed.

### Rheumatoid Arthritis

Rheumatoid arthritis (RA) is a chronic systemic autoimmune disease that primarily affects the lining of the synovial joints, resulting in both cartilage destruction and bone erosion. The disorder may lead to progressive disability and premature death, contributing to a significant clinical and economic burden on society (60). Some biologic agents such as cytokine antagonists that inhibit TNF-α, IL-1β, or IL-6 (anti-TNF-α, anti-IL-1, or anti-IL-6) have shown preventive effects that can substitute for conventional disease-modifying anti-rheumatic drugs (61). However, no clinically useful biologic therapy is available for the individually tailored treatment of RA, and patients with RA usually require a long-term treatment plan, in which side effects are generally inevitable (62). Thus, the development of an innovative prophylactic or therapeutic strategy is critical, and there has been considerable interest in developing recombinant immune-modulating drugs for RA treatment.

### Collagen-Induced Arthritis Autoimmune Mouse Model for Rheumatoid Arthritis

The CIA autoimmune mouse model is widely used to study RA (63, 64). Infection of mice with either *S. mansoni* or *S. japonicum* prior to collagen immunization reduced the severity of CIA (65–67). In CIA mice, a prior *S. mansoni* infection was found to down-regulate the splenic production of Th1 (IFN-γ), and pro-inflammatory cytokines (TNF-α, and IL-17A), and this was accompanied by the up-regulation of anti-arthritic cytokines (IL-4 and IL-10) (65). Subsequently it was demonstrated that the protective effect of a *S. japonicum* infection against CIA is infection stage-dependent, i.e., protection was only provided in the ASCIA group {when the first injection of type II collagen (CII) was given at the acute stage [7 weeks post infection (p.i.)]}, but not in the ESCIA group [when the first injection of CII was given at an early stage (2 weeks p.i.)] (66). The protective effects in the former group were associated with increased production of IL-4 and IL-10 and reduced production of IFN-γ in the spleen. As a result, the importance of a dominant and long-lasting Th2 response in suppressing autoimmune joint inflammation was suggested by these authors (66). Another group then explored the effects of *S. japonicum* infection on CIA by challenging DBA/1 mice with unisexual or bisexual cercariae 2 weeks prior to CII injection or at the onset of CIA (67). This study showed that *S. japonicum* infection (unisexual or bisexual) 2 weeks prior to CII immunization significantly reduced the severity of CIA. This outcome was consistent with earlier results obtained with a *S. mansoni* infection by Osada et al. (65) but not with previous observations by He et al. (66). In the protected mice, significant down-regulation of Th1 (IFN-γ) and pro-inflammatory cytokines (TNF-α, IL-1β, and IL-6), and up-regulation of Th2 (IL-4) and the anti-inflammatory cytokine IL-10 were observed. Song et al. (67) also demonstrated that when the established CIA mice were challenged with bisexual *S. japonicum* cercariae, exacerbating effects on the disease were elicited at 1-week p.i. Notably, *S. mansoni* infection exhibited both ameliorating and exacerbating effects on spontaneous autoimmune arthritis in IL-1 receptor antagonist (IL-1Ra)-deficient mice (68). While *S. mansoni* infection partially protected the IL-1Ra-deficient mice from arthritis with reduced IL-17 and TNF-α and enhanced IL-4 and IL-10 splenic responses, the infected mice had increased levels of IgG rheumatoid factor and anti-dsDNA IgG in serum which likely contributed to the exacerbating autoimmune effects (68). By employing signal transducer and activator of transcription 6 (STAT6) knock-out (KO) and IL-10 KO mice, Osada et al. (31) later demonstrated that STAT6-related cytokines (IL-4, IL-13) and IL-10 are essential for the suppression of CIA by a *S. mansoni* infection.

### Adjuvant Arthritis (CFA-Induced AA) Induced Rat Model

Schistosome antigenic components have been employed to circumvent the potential deleterious effects caused by a live worm infection. Autoclaved antigen, derived from *S. mansoni* cercariae (ASMA), has been tested for a potential protective effect on adjuvant arthritis (CFA-induced AA) induced in rats by subcutaneous and intradermal injections of complete Freund’s adjuvant into the paw and tail, respectively (32). Intradermal injection of ASMA, after CFA-induced AA, attenuated the progression of clinical signs of polyarthritis, and improved the gait and increased the body weight of animals, with reduced production of IL-17 and increased serum levels of both IL-10 and IFN-γ. The authors suggested that up-regulation of Foxp3^+^ Tregs, with subsequent modulation of both pro- and anti-inflammatory cytokines, contribute to the anti-arthritic activity (32). Unlike the CIA model, in which it has been proposed that the anti-arthritic effect of schistosome infection is induced by Th2-polarization with increased levels of protective Th2 cytokines and suppression of the pathogenic Th1 cytokines, in the CFA-induced AA rat model, IFN-γ (the cytokine strongly associated with a Th1 response) increased after ASMA treatment; this may have been due to the presence of mycobacteria, constituting the main antigenic component in the CFA adjuvant, inducing the high level of endogenous IFN-γ recorded (32). Nevertheless, these authors concluded that in this case, the higher level of IFN-γ might have contributed to ameliorating rather than exacerbating the effects (32).

### rSjCystatin and SJMHE1 as Immunomodulators

To date, two immunomodulatory molecules originating from *S. japonicum* have shown prophylactic/therapeutic effects on CIA in the murine model. Prophylactic injection of rSjCystatin, i.e. administration prior to CIA, was shown to significantly alleviate tissue pathologies based on parameters such as paw clinical scores, incidence of arthritis, and histopathology scores of joints (33). These effects appeared to be related to the inhibitory modulation of Th1 and Th17 responses and the upregulation of Th2 and Tregs responses as evidenced by a shifted cytokine profile, i.e. the levels of anti-arthritic cytokines (IL-4 and IL-10) were notably increased, while the levels of IFN-γ, and pro-inflammatory cytokines (IL-6, IL-17, and TNF-α) were significantly suppressed in the prophylactic rSjCystatin-treated mice (33). In contrast, therapeutic injection of rSjCystatin, i.e. administration of rSjCystatin to mice with established CIA, showed no significant differences in clinical parameter incidence and histological examination scores, suggesting that post-injection of rSjCystatin does not prevent the outbreak of inflammation or synovitis and cartilage degradation in these mice (33). Treatment with SJMHE1, a short linear peptide from the HSP60 protein of *S. japonicum*, resulted in a significant reduction in joint inflammation, accompanied by suppressed clinical symptoms, lower incidence of arthritis and reduced severity of arthritis in CIA mice (34). In this study, SJMHE1 injection significantly reduced inflammation, pannus, and cartilage and bone damage scores on histopathological examination of mouse paws. Similar to that observed with rSjCystatin, these effects were linked to the modulation of key cytokines involved in the pathogenesis of CIA, wherein the splenic expression levels of IFN-γ, IL-22, and pro-inflammatory cytokines (TNF-α, IL-6, and IL-17) were down-regulated while the inhibitory cytokine IL-10, TGF-β1 mRNA, and the percentage of CD4^+^CD25^+^Foxp3^+^ T cells (Tregs) were up-regulated (34).

### Therapeutic Effects of Sj16 and SmKI-1

Additional individual *Schistosoma*-derived molecules have also been shown to exhibit therapeutic effects on arthritis models such as the CFA-induced AA rat model and the monosodium urate (MSU)-induced gout arthritis model. Treatment with rSj16, a 16-kDa recombinant protein from *S. japonicum*, induced an anti-inflammatory effect, and was shown to protect rats from CFA-induced knee joint inflammation and paw swelling in a dose-dependent manner, with the serum levels of TNF-α, NO, and IL-1β decreased and IL-10 increased (69). These effects were thought to be associated with interruption of maturation and function of DCs, in an IL-10-dependent manner (69). The N-terminal nuclear localization signal (NLS) domain of rSj16 has been demonstrated to be associated with increased production of IL-10 (70). Another molecule of interest is SmKI-1, a Kunitz type protease inhibitor from *S. mansoni* (71) which plays an important role in inhibiting neutrophil function (35). Mice given rSmKI-1 intravenously showed decreased inflammation in the knee joint after monosodium urate (MSU) administration with a 90% decrease in myeloperoxidase (MPO) activity, and reduced neutrophil accumulation, hypernociception, and overall pathological score (35). In addition, rSmKI-1 significantly decreased the level of the pro-inflammatory cytokine IL-1β in MSU-induced gout, promoting neutrophil influx to the sites of tissue injury and those associated with joint damage. Joint damage was also reduced with diminished leukocyte infiltration and hyperplasia from the synovial membrane after rSmKI-1 treatment. Similar to rSmKI-1, an active *S. mansoni* infection also exhibited modulating effects on MSU-induced gout arthritis with a significant reduction in neutrophils in the articular knee cavity (35).

### Allergy and Asthma

The prevalence of these two atopic diseases has increased dramatically over the past decades worldwide, not only in developed but also in developing countries (72). The immunopathogenesis of allergic sensitization, such as occurs in atopic asthma, is associated with cytokines produced by Th2 cells, including IL-4, IL-5, IL-9, and IL-13 (73). It has been speculated, based on epidemiological and experimental evidence, that helminth infections or their products may help control the development of allergy and asthma. *S. mansoni* has been shown to be highly effective in protecting humans and mice against allergic sensitization (73, 74). However, the association between schistosome infection and allergic disease is still elusive due to conflicting results of epidemiological studies in low-income countries, with some reports showing an inverse association between schistosome infection and allergic diseases, while others recorded positive schistosome-allergy associations including mite atopy in Ghanaian schoolchildren (75), atopy and wheeze in Uganda fishing communities (76), and allergy in Zimbabweans (77). A cross-sectional study in a schistosomiasis-endemic area in Brazil revealed an inverse association between *S. mansoni* infection burden and allergic reactivity to common household dust allergens in individuals eliminating more than 12 eggs/g of feces (78). Overall, these studies indicate that the effect of a schistosome infection on allergic disease is complex, involving multiple factors including genetic associations, infection burden, and the presence of co-infections.

### Ovalbumin/Alum-Induced AAI Model for the Study of Allergic Asthma

Over the past two decades, there has been considerable interest in exploring the effect of schistosome infection (79–81) or schistosome-derived products (82–84) on the development of airway inflammation in different mouse models. A widely used murine model is the OVA/alum-induced AAI model for the study of allergic asthma, a chronic inflammatory airway disease characterized by reversible airflow obstruction, which represents over 60% of all asthma case (85). Another important model is the house dust mite (HDM, *Dermatophagoides pteronyssinus*, *Der p1*)-induced allergic airway inflammation model, mimicking severe asthma, in which Th17 and neutrophils are dominant responders (41).

Though both schistosomiasis and allergic diseases such as asthma can stimulate a strong Th2-type immune response with elevated concentrations of IgE and eosinophilia, schistosome infection can also reduce the inflammation due to allergic asthma by modulating a variety of immune cells, cytokines and chemokines resulting in a decreased Th2 response. For example, chronic *S. japonicum* infection suppressed airway eosinophilia, mucus production and antigen-specific IgE responses induced by OVA sensitization and challenge with reduced allergen-driven IL-4 and IL-5 production, but had no significant effect on IFN-γ production (86). In this study, dendritic cells were suggested to be involved in the process of helminth infection-mediated modulation of allergic inflammation with a significant decrease in IL-4/IL-5 production and increased IL-10 production (86). In the HDM-induced murine asthma model, *S. japonicum* infection prior to modeling significantly attenuated airway hyper-responsiveness by reducing the infiltration of inflammatory cells (particularly eosinophils and neutrophils) into the bronchoalveolar lavage (BAL) fluids and the lungs, during the early and late stages of HDM sensitization and challenge (41). These effects were associated with the down-regulation of the Th2 cytokine IL-4, which inhibited eosinophil infiltration and the Th17 cytokine IL-17, which contributed to the amelioration of neutrophil infiltration; in contrast, comparable levels of the Th1 cytokine IFN-γ were observed in the BAL fluids and lungs of infected and uninfected mice indicating limited modulation of the Th1 response (41). This protective mechanism in allergic airway inflammation may be related to the continuous up-regulation of Treg cells in the spleen upon *S. japonicum* infection (41). In addition, *S. japonicum* infection causes the down-regulation of serum HDM-specific IgE that can activate mast cells and basophils during the immunopathogenesis of asthma, and is associated with reduced allergic airway inflammation (41). Infection with *S. mansoni* prevents allergic airway inflammation and anaphylaxis in mice through the induction of IL-10-producing CD1d(high) regulatory B cells that were shown to prevent ovalbumin-induced allergic airway inflammation following passive transfer to ovalbumin-sensitized recipients; the regulatory B cells induced pulmonary infiltration of Tregs, independent of TGF-β, thereby suppressing allergic airway inflammation (87).

Similar to a live worm infection, intraperitoneal injection of isolated *S. mansoni* eggs prior to allergic sensitization also showed a protective effect on OVA/alum-induced AAI with less eosinophilia in the BAL and lungs, less cellular influx into lung tissue, less allergen-specific Th2 cytokines (IL-5 and IL-13) in bronchoalveolar lavage fluid (BALF) and mediastinal lymph nodes, and lower levels of OVA-specific IgG1 and IgE antibodies in serum (36). Notably, although an allergic OVA-specific Th2 response was absent, treatment with egg antigens induced a strong systemic egg‐specific Th2 response with increased levels of IL-5, IL-13, and IL-10. Additionally, the *S. mansoni* egg‐induced protection was independent of both Tregs and B cells, but was associated with reduced pulmonary influx of pro-inflammatory monocyte-derived dendritic cells (moDCs) (36).

In a murine model of asthma, treatment with soluble schistosome egg antigens (SEA) significantly increased the percentage and suppressive activity of regulatory CD4^+^CD25^+^ T cells, inhibited the expression of Th2 cytokines (IL-4 and IL-5), relieved antigen-induced airway inflammation, and suppressed asthma (82). Recently, Marinho et al. (37) demonstrtaed the ability of the *S. mansoni* schistosomula tegument (Smteg) to modulate OVA-induced airway inflammation in a murine model. Treatment with Smteg during OVA sensitization resulted in a reduction of protein extravasation and the number of eosinophils in the BAL, and decreased inflammation, collagen deposition and also eosinophil numbers in the lungs. Pro-inflammatory cytokines and chemokines (IL-5, IL-13, IL-25, and CCL11) and specific anti-OVA IgE levels were decreased and the levels of IL-10 were increased in the lungs. However, IL-10 was shown to be produced by monocytes with a significantly increased percentage of CD11b^+^F4/80^+^IL-10^+^ and CD11c^+^CD11b^+^IL-10^+^ cells in the lungs of Smteg-treated mice. Overall, this study suggested that the modulation of Smteg occurred mainly through the activity of macrophages and DCs but not Th cells, and emphasized the role of innate immunity over adaptive immunity in airway inflammation in Smteg-treated animals (37).

### Sm22.6, PIII, and Sm29 as Immune-Modulators in the Murine Model of OVA-Induced AAI

A number of other *Schistosoma*-derived molecules have been tested for their ability to suppress allergic airway inflammation. For example, Cardoso et al. (83) explored the capability of three *S. mansoni* antigens, Sm22.6, PIII, and Sm29, in modulating the immune response in the murine model of OVA-induced AAI. Immunization with these *S. mansoni* antigens protected mice against allergic inflammation, as evidenced by a significantly reduced number of inflammatory cells and eosinophils being recruited to the airways, and the reduced serum level of OVA-specific IgE produced in the immunized animals. The frequency of Tregs was higher in the groups of mice immunized with Sm22·6, Sm29 and PIII, compared with controls, but higher levels of IL-10 in the BAL relative to the non-immunized group was observed only in mice immunized with Sm22·6; decreased levels of IL-4 and IL-5 in the BAL were observed in mouse groups immunized with PIII and Sm22·6 compared with non-immunized animals. Collectively, the study by Cardoso et al. (83) implied that Tregs might play a key role in this process of immune modulation. However, different insights were obtained with Sm29 in a human study by de Almeida et al. (38), in which by employing peripheral blood mononuclear cells (PBMC) isolated from asthmatic patients, Sm29 and Sm29TSP-2 (a chimeric antigen comprised of Sm29 and SmTSP-2) were tested for their ability to modulate lymphocyte activation in response to the house dust mite allergen *Der p1*. The addition of both antigens to PMBC cultures from asthmatic subjects stimulated with *Der p1* showed an increased frequency of CD4^+^CD25^hi^ T lymphocytes and a decreased frequency in the population of CD4^+^CD25^low^ cells compared with unstimulated groups; in contrast, no significant difference was observed in the frequency of CD4^+^CD25^hi^ T cells expressing Foxp3 in the cultures stimulated with *Der p1* in the presence or absence of either antigen (38).

### Schistosome Peptides Can Modulate Immune Responses in Airway Inflammation

Peptides from *S. japonicum* have also shown potential to modulate the immune response in airway inflammation by down-regulating Th2 and up-regulating Th1 response and Tregs and IL-10. For example, immunization with peptides P6, P25, and P30 from SjP40, a *S. japonicum* protein homologue of SmP40 (the major egg antigen in *S. mansoni*), was shown to inhibit airway inflammation in the OVA-induced allergic asthma mouse model, with reduced peribronchial airway inflammation and mucus production, decreased airway cellular infiltration and a substantial reduction in eosinophils in bronchoalveolar lavage fluid (BALF) (39). Compared with OVA only treated mice, the SjP40 peptides induced production of Th1-type cytokines, particularly IFN‐γ, and inhibited the production of Th2 cytokines, such as IL‐4, IL‐5, and IL‐13 (39). In addition, immunization with the three peptides resulted in an increased level of IgG2a, promoted by IFN‐γ, but decreased IgE and IgG1 antibody production, promoted by Th2 cytokines. These data revealed a novel immune protective mechanism involving T cell epitopes from helminth-derived Th1-inducing antigens in modulating allergic asthmatic responses through an enhancing Th1 response (39). Also, the aforementioned peptide, SJMHE1, suppressed airway inflammation in the OVA-induced allergic asthma mouse model with modulation of pro- and anti-inflammatory cytokines in splenocytes and lungs, and decreased numbers of infiltrating inflammatory cells and eosinophils (40). SJMHE1 treatment during OVA sensitization and challenge in BALB/c mice significantly reduced the IL‐4 mRNA level in the splenocytes, suppressed the expression of IL‐4, IL‐5, and IL‐17 mRNA in the lungs, and increased IFN‐γ, IL‐10, and IL‐35 mRNA, indicating the suppressive effect was likely associated with decreased populations of Th2 and Th17 cells and an increased frequency of Th1 and Treg cells (40). While the suppressive mechanism involving the elevation of IL-10 in the allergic asthma mouse is different from that in the CIA model, schistosomes and their products modulate distinct targets regulating Th1/Th2/Th17-associated inflammation in the different models. SJMHE1 treatment did not alter IgE levels, suggesting that application of small peptide molecules from schistosomes and other helminths as potential drugs for allergic asthma or other allergic diseases may represent a safer therapy pipeline compared with utilizing a live worm infection or whole parasite proteins, which may elicit unwanted side effects in patients.

### Inflammatory Bowel Disease *-*Like Colitis

Inflammatory bowel disease (IBD) produces symptoms such as diarrhea, fatigue, bloody stool, and weight loss; it is a chronic inflammatory disorder of the gastrointestinal tract and mainly includes Crohn’s disease (CD) and ulcerative colitis (UC) (88). Although the pathogenesis of IBD is still not completely clarified, the disease is suggested to be caused by an uncontrolled aggressive cellular immune response in genetically prone individuals (89). The inflammation during CD is associated with Th1 lymphocytes and Th17 cells (90); UC is associated with an atypical Th2 response characterized by antigen in the mucosal microfiora activating NKT cells that, in turn, secrete IL-13, resulting in the cytolysis of epithelial cells (91). A recent epidemiological study showed that the prevalence of IBD is consistently higher in countries with a high socio-demographic index such as the UK, the USA, Canada, and Australia (92).

### IBD-Like Colitis Models

Two types of IBD-like colitis murine models have been developed for the study of immune-regulating mechanisms of helminth infections on IBD. One is a chemical-induced experimental colitis model in which there is interference of the intestinal mucosa by administration of dextran sulfate sodium (DSS), or di- or tri-nitrobenzene sulphonic acid (DNBS or TNBS). This type of model can be used for exploring the involvement of innate immune cells such as DCs, eosinophils and macrophages in immune-regulating mechanisms (48, 93). The other type gives rise to an inflammatory T cell response, an example being the T-cell transfer model, which is used for investigating the modulatory mechanism of the adaptive immune system on IBD (45).

Infection with *S. mansoni* male worms reduced the severity of DSS-induced colitis in BALB/c mice, but neither a male-female paired egg-laying worm infection nor the injection of eggs was effective (93). The authors suggested that the protection from colitis by a male-worm only infection was mediated by a mechanism dependent on a novel colon-infiltrating macrophage population (F4/80^+^CD11b^+^CD11c^-^) but not on Tregs and other lymphocytes or a simple modulation of Th2 responses. In contrast, egg-laying worms in outbred NMRI mice attenuated some clinical symptoms of colitis such as body weight loss and shortening of colon length when DSS was administrated 9 weeks p.i (94). Furthermore, Floudas and co-workers (43) compared the fecal microbiota of mice infected with adult male *S. mansoni* worms, and male- and female-worm-infected mice and showed that schistosome infection altered the composition of the intestinal microbiota which in turn modulated susceptibility to DSS-induced colitis. In this study, mice with a *S. mansoni* male-only worm infection showed reduced susceptibility to colitis after DSS administration with a significant decrease in the disease activity index (DAI) score, colon damage and MPO activity, while a *S. mansoni* male-female worm infection exacerbated the severity of colitis. Compared to uninfected mice, *S. mansoni-*infected individuals harbored changes in microbial species with a significant increase in colitogenic microbiota such as *Parabacteroides* and *Bacteroides* genera, which are associated with aggravated experimental colitis. In addition, the male-female-infected mice had a distinct microbiota composition compared to that of male-infected mice and uninfected controls, which may influence the development of colitis (43). *S. japonicum* infection also showed potential for attenuating DSS-induced colitis in Kunming mice, a feature which has been linked to decreased Th1, Th2 and Th17 responses, characterized by a significant decrease in the serum levels of IL-6, IL-2, IL-10, IL-17, IFN-γ and TNF-α (42). In detail, after administration with DSS at 4 weeks p.i., *S*. *japonicum*-infected mice had longer colon lengths, suffered less weight loss, lower histological and DAI scores, and less infiltration of inflammatory cells into colon tissue compared with uninfected DSS-treated mice. Moreover, the expression levels of proteins involved in endoplasmic reticulum (ER) stress, such as IRE1α, IRE1β, GRP78, and CHOP, were lower in the *S. japonicum*-infected and DSS treated group compared with the DSS alone group, indicating ER stress could be involved in attenuating DSS-induced colitis in mice after exposure to *S. japonicum* (42).

With TNBS-induced colitis, which shares features of CD, *S. mansoni* infection (95), injection of freeze/thawed-killed eggs (from both *S. mansoni* and *S*. *japonicum*) (96–98), or *S. mansoni* soluble worm proteins (SmSWP) (90) attenuated inflammation in experimental animal models. *S. mansoni* infection, prior to intracolonic administration of TNBS, significantly reduced the severity of the TNBS-induced immune disorder in a rat model, as evidenced by lower macroscopic and microscopic damage scores and by a more rapid decrease in colonic MPO activity compared with injection of TNBS alone (95). Exposure to schistosome eggs ameliorated TNBS-induced gut inflammation in mice by reducing the Th1 response and increasing the Th2 response, as shown by decreased IFN-γ but increased IL-10 expression in a variety of tissues such as colon and spleen and in serum (96–98). In addition, the percentages of Tregs increased in the spleens of egg-exposed and TNBS-treated mice compared with TNBS-treated animals alone (97). In a study exploring the therapeutic potential of helminth soluble proteins in TNBS-induced colitis, treatment with SmSWP suppressed the expression of pro-inflammatory cytokines (IFN-γ and IL-17) in the colon and mesenteric lymph nodes, whereas there was a significant increased production of regulatory cytokines (IL-10, TGF-β) in colon tissue (90). These observations suggest that the protective effects of schistosome infection or schistosome products in TNBS-induced colitis are linked to increased Th2 and Treg responses.

With respect to SEA, the immunomodulation effects are dependent on the timing of antigen injection and DSS administration. Injection with SmSEA after the commencement of DSS application did not protect NMRI mice from colitis (94). In contrast, SmSEA immunization prior to DSS application markedly ameliorated the course of DSS-induced colitis characterized by lower DAI and macroscopic inflammatory scores, reduced MPO activity, and increased expression of FoxP3^+^ Tregs and Th2 cytokines, suggesting that SmSEA may have potential for development as a prophylactic helminthic therapy due to this positive modulatory effect (44). However, these observations conflict with an earlier report by Smith et al. (93) showing that an egg-laying schistosome infection or injection of eggs did not render mice resistant to colitis induced by DSS. In an adoptive T-cell transfer SCID mouse model, induced by transfer of CD4^+^CD25^-^CD62L^+^ T cells, SmSEA alleviated the severity of colitis through a colonic T-cell-dependent mechanism (45). Repeated administration of SmSEA weekly or twice a week ameliorated clinical signs and intestinal inflammation with significant reductions in the clinical disease and colonoscopic scores. Twice a week administration of SmSEA induced the anti-inflammatory effect of a Th2 response, which down-regulated the number of IL-17a-producing effector T cells (Th17 cells) and significantly upregulated the number of IL-4-producing effector Th2 cells in the colonic lamina propria mononuclear cells (LPMCs) (45).

### Schistosoma Recombinant Proteins and Extracellular Vesicles for Treating IBD-Like Colitis

Recently, *Schistosoma*-derived recombinant proteins and extracellular vesicles (EVs), especially exosome products from innate immune cells stimulated by schistosome antigen, also exhibited therapeutic potential for the treatment of IBD-like colitis. Recombinant Sj16 (rSj16), a 16-kDa secreted protein of *S*. *japonicum* produced in *Escherichia coli*, was shown to alleviate disease severity in DSS-induced colitis mice; this resulted from down-regulation of pro-inflammatory cytokines such as TNF-α, IFN-γ, IL-17a, and Chil3, whose expression is high in IBD individuals, and up-regulation of the anti-inflammatory cytokines (TGF-β and IL-10), with increased percentages of Tregs (46). Moreover, the treatment of rSj16 on DSS-induced colitis altered the expression of specific genes in the colon, leading to the inhibition of the PPAR-α signaling pathway which plays an important role in the development of DSS-induced colitis (46).

Injection of recombinant *S*. *japonicum* cystatin (rSjcystatin) after TNBS administration significantly reduced inflammatory parameters and ameliorated the severity of colitis in mice; this resulted from a decreased level of IFN-γ in three organs and elevated levels of IL-4, IL-13, IL-10, and TGF-β in the colon and increased numbers of Tregs in the mesenteric lymph nodes (MLNs) and intestinal lamina propria mononuclear cells (LPMCs) (47). However, injection of rSjcystatin prior to TNBS induction failed to show decreases in the inflammation indexes compared with the colitis mice (47).

Unlike rSjcystatin, administration of *S. haematobium* glutathione S-transferase (P28GST), a recognized vaccine candidate against urinary schistosomiasis, prior to TNBS induction, exhibited a more beneficial effect on the modulation of disease severity and immune responses in experimental colitis (48). Immunization of rats or mice with P28GST showed an anti-inflammatory effect at the same level as schistosome infection in reducing acute colitis and the expression of pro-inflammatory cytokines (48). P28GST induced a Th2 response involving increased eosinophil infiltration suggesting that eosinophils play a crucial role in the immunomodulation of colitis by P28GST (Driss et al., 2016). Furthermore, P28GST, applied in the form of biodegradable and biocompatible poly(lactic-co-glycolic acid) (PLGA)-based microparticles before TNBS induction, showed potential for preventive treatment with the Wallace score (a measure of the severity of inflammation) being significantly decreased in mice compared with a placebo (99).

In another approach, Wang et al. (100) demonstrated that intraperitoneal injection of mice with exosomes derived from DCs treated with *S*. *japonicum* SEA alleviated established acute DSS-induced colitis. Compared with SEA-untreated DC exosomes and SEA, SEA-treated DC exosomes showed a greater effect in alleviating the clinical scores on body weight loss, diarrhea and bleeding, and also, prevented colon damage and ameliorated the reduction in colon length; pro-inflammatory cytokines, TNF-α, IFN-γ, IL-17a, IL-12, and IL-22, were decreased after the treatment, but the precise mechanism involved needs to be further investigated.

### Type 1 Diabetes

Type 1 diabetes (T1D) is an organ-specific autoimmune disorder caused by the immune system attacking and destroying insulin-producing β cells in the pancreas (101, 102). The prevention of the disease in individuals at-risk has proved challenging. The decreased exposure to helminths in modern societies has been suggested as a key factor involved in the raised incidence of T1D. Indeed, it has been proposed that the hygiene hypothesis should be extended from allergic to autoimmune diseases as well (24). T1D is recognized mainly as a Th1‐mediated disorder, although recent data indicate the possible involvement of follicular helper T cells, as well as T cells co‐producing IFN‐γ and IL‐17 during the development of the disease (103). The immunomodulatory mechanisms in helminth infections that protect against T1D include a Th1 to Th2 shift and Tregs expansion (104).

Cooke et al. (105) were early pioneers in the field exploring the preventive effect of schistosome infection and schistosome products on T1D. They found that *S. mansoni* infection and eggs prevented T1D in non-obese diabetic (NOD) mice, with the immune response switching from Th1 to Th2 (105). Furthermore, injected soluble extracts of *S. mansoni* worms or eggs were shown to completely prevent the onset of T1D in the NOD mouse but only when the administration commenced in animals at 4 weeks of age, with potential involvement of the innate immune system, and cellular participation involving bone marrow‐derived DCs and Vα14*i* NKT cells (106). The same group demonstrated that SmSEA prevented T1D onset by enhancing Th2 responses and Treg activity in NOD mice, and suggested that TGF-β from T cells is crucial in the prevention of T1D (29, 107). At the molecular level, ω-1, a well-characterized glycoprotein in SEA responsible for inducing a Th2 response, was identified as a key component involved in the induction of Foxp3^+^ Tregs in NOD mice (108). Recently, the aforementioned protein, rSjCystatin, which is a secretory cysteine protease inhibitor, and recombinant fructose-1,6-bisphosphate aldolase from *S*. *japonicum* (rSjFBPA) exhibited potential to significantly reduce the onset of T1D and ameliorate its severity in NOD mice; this was associated with increased production of Th2 and Treg cytokines, such as IL-10 and TGF-β (49). In streptozotocin (SZT)-induced diabetic mice, *S. mansoni* infection or SmSEA also proved to be protective against the disease (109, 110). Furthermore, another study showed that *S. mansoni* infection could partially protect pancreatic islets from degradation and induced an anti-hyperglycemia effect in STZ-induced experimental T1D mice, that was independent of T-cell cytokine modulation (IL-10), STAT6 and Tregs (50).

### Type 2 Diabetes

Type 2 diabetes (T2D) is a common inflammatory disease characterized by persistent hyperglycemia due to insulin resistance. It has been estimated that the incidence of diabetes was 463 million in 2019, and the figure may increase to 700 million by 2045, with approximately 90% of cases being due to T2D (111). The regulatory mechanisms through which *Schistosoma* infection and *Schistosoma* products modulate the innate and adaptive immune responses against T2D have been comprehensively reviewed (30). In a cross-sectional study, Chen et al. (112) reported that T2D prevalence was lower in individuals with a previous *S. japonicum* infection (PSI) than those without a PSI (14.9% vs. 25.4%); PSI was also associated with a lower body mass index, and reduced blood glucose, glycated hemoglobin A1c, and insulin resistance score. It has also been shown that *S. mansoni* infection and SmSEA protect against metabolic disorders such as T2D by promoting a Th2 response, eosinophilia, and white adipose tissue (WAT) M2 (alternatively activated macrophages) polarization (51). Similarly, using a type 2 diabetes *Lep*r*^db/db^* mouse model, it was demonstrated that the administration of 50 µg *S. japonicum* SEA twice a week for 6 weeks significantly reduced insulin resistance and blood glucose and correlated with an elevation in the level of the Th2 cytokines IL-4 and IL-5 in spleen cells (52). Furthermore, the frequency of spleen regulatory T cells increased significantly in the SEA-administrated group, suggesting key roles for Th2 and Treg responses induced by SEA in reducing insulin resistance; SEA can thus provide a potential novel therapy for the treatment of T2D (52).

### Sepsis

Despite the availability of modern antibiotics and resuscitation therapies, sepsis remains one of the major threats to critically ill patients in terms of morbidity and mortality (113). Currently, a new consensus definition for sepsis has been recommended; that it is a life-threatening organ dysfunction caused by a dysregulated host response to infection (114). Microvascular damage occurs in the early stage of sepsis and can lead to multisystem organ dysfunction (MODS) and ultimately death (115). The inflammatory response elicited by schistosomal eggs trapped in the intestinal wall facilitates their movement from the vascular system to the gut, which may result in the simultaneous translocation of bacteria. It has been demonstrated that SmSEA exhibits a suppressive effect on dendritic cells in the inflammatory response to pathogenic factors, such as lipopolysaccharide (LPS), CpG, and poly-I:C, which can lead to sepsis (116). Furthermore, *S. japonicum* infection can activate macrophage differentiation into the M2 phenotype and suppress LPS-induced M1 macrophage activation in the LPS-induced septic mouse model (53). Recently, Li et al. (54) investigated the role of *S*. *japonicum* cystatin (rSj-Cys) in regulating the inflammatory response of bacterial sepsis induced in BALB/c mice by cecal ligation and puncture (CLP). Treatment with rSj-Cys provided significant therapeutic effects on CLP-induced sepsis in the mice characterized by increased survival rates, alleviated overall disease severity with reduced tissue injury in the kidney, lung and liver. These outcomes were associated with upregulated levels of IL-10 and TGF-β1 cytokines and reduced pro-inflammatory cytokines IL-1β, IL-6, and TNF-α; MyD88 expression in liver, kidney and lung tissues of rSj-Cys-treated mice was reduced. *In vitro* assays also showed that rSj-Cys inhibited the release of mediator nitric oxide and pro-inflammatory cytokines by macrophages stimulated by lipopolysaccharide (LPS). These therapeutic effects were thus associated with downregulation of pro-inflammatory cytokines and upregulation of regulatory cytokines (54). The same team showed that in a murine model of LPS-induced sepsis, intraperitoneal administration of rSj-Cys significantly alleviated LPS-induced organ pathologies, reduced the levels of liver and renal functional indexes and pro-inflammatory cytokines, and increased the serum level of IL-10 (55). The same group showed that rSj-Cys significantly reduced sepsis-induced cardiomyopathy in mice and suggested this molecule should be considered as a potential therapeutic for preventing and treating sepsis-associated cardiac dysfunction (117).

### Cystitis

Chronic cystitis, an inflammation of the urinary bladder, often due to bacterial infection, is also a feature of urogenital schistosomiasis caused by *S. haematobium* (118). Unexpectedly, some molecules derived from this schistosome species have been shown to have potential in the treatment of cystitis. A major *S. haematobium* egg secretory protein H-IPSE [the homolog of IL-4-inducing principle of *S. mansoni* eggs (M-IPSE)], which can infiltrate the nuclei of host cells and bind genomic DNA (119), has been found to alleviate chemotherapy-induced hemorrhagic cystitis (CHC) in a mouse model *via* the down-regulation of pro-inflammatory pathways including the IL-1β-TNFα-IL-6 pathway, interferon signaling, and a reduction in oxidative stress (56, 120). It was shown that a single intravenous dose of H-IPSE (H-IPSE^H06^) given to mice was more effective than 2-mercaptoethane sulfonate sodium (MESNA), the current drug of choice for mitigating CHC, in an IL-4-dependent manner (120). This study represents the first therapeutic exploitation of a uropathogenic-derived molecule in a clinically relevant bladder disease model (120). In a subsequent study, the same group found that local bladder injection of the IPSE ortholog, H-IPSE^H03^, might be more effective in preventing hemorrhagic cystitis than the systemic administration of IPSE^H06^ (121).

### Cancer

A substantial body of evidence supports the association between long-standing chronic inflammation and cancer (122). Schistosomiasis and the liver fluke diseases opisthorchiasis and clonorchiasis can induce carcinogenesis (123). Indeed, *S. haematobium* is a Group-1 carcinogen and, alarmingly, is the leading cause of bladder cancer globally (124). On the other hand, there is growing evidence showing that parasite infection or parasite-derived products can also reduce cancer tumorigenesis through the induction of apoptosis, activation of the immune response, avoidance of metastasis and angiogenesis (125, 126). For the first time, *S. mansoni* was reported recently to have a therapeutic effect on murine colon cancer (57). *S. mansoni* antigen was shown to inhibit colon carcinogenesis with significant decreases in tumor lesion size and the number of neoplasias; although the antitumor mechanism operating remains to be determined, this study suggests that schistosome antigens could potentially play a role in future cancer treatment (57).

It is noteworthy that recent research showed that schistosome-derived miRNAs can also mediate anti-tumor activity in host liver cells during schistosome infection (58). An *S. japonicum* egg EVs-derived miRNA (Sja-miR-3096) was shown to be present in the hepatocytes of infected mice. The miRNA significantly prevent the growth of liver tumor cells by cross-species regulation of the murine and human phosphoinositide 3-kinase class II alpha (*PIK3C2A*) gene. The Sja-miR-3096 mimics suppressed cell proliferation and migration of both human and murine hepatoma cell lines by targeting phosphoinositide 3-kinase class II alpha (PIK3C2A). A murine hepatoma cell line was generated that stably expressed the pri-Sja-miR-3096 gene and cross-species processing of the schistosome pri-miRNA to the mature Sja-miR-3096 in the mammalian cell was demonstrated; inoculation of this cell line into the scapula and livers of mice led to the complete suppression of tumorigenesis of the hepatoma cells. In addition, tumor weight was significantly reduced after intravenous administration of Sja-miR-3096 mimics. Thus, schistosome miRNA-mediated anti-tumor activity occurs in host liver cells during schistosomiasis, thereby increasing host resistance to liver cancer, and points the way forward to developing parasite miRNAs as promising new agents for cancer treatment (58).

## Current Challenges and Future Perspectives

Despite recent key findings using active schistosome infection or *Schistosoma* components for the treatment of autoimmune and inflammatory diseases, cancer, and other illnesses, some challenges remain and these and future perspectives are now considered.

(1) A number of clinical trials with controlled helminth infections have been applied in the treatment of inflammatory diseases with disappointing and conflicting results (127). So far, no similar work has been carried out with controlled schistosome infection. However, a controlled human *S. mansoni* infection model now has been estalished (128), which may not only advance the development of novel therapeutics, diagnostics and vaccines for schistosomiasis, but may also pave the way for controlled human schistosome infection studies for the treatment of autoimmune and inflammatory diseases.

(2) Most reports and observations in this area are based on murine models; while extensively providing insights and evidence to predict the utility of schistosome molecules for the treatment/alleviation of human diseases, the suitability of the mouse to recapitulate human conditions remains in question for immune-mediated inflammatory diseases, since there are considerable differences between human and mouse immunology (129). In addition, there are considerable differences in the patterns of gene expression associated with inflammatory diseases in human patients compared with animal models (130). Thus, future studies will need to change focus from studies on animal models to undertaking human clinical trials.

(3) A limited number of schistosome molecules have been investigated for potential in treating autoimmune and inflammatory diseases. In-depth transcriptomic and proteomic analysis of the different schistosome species and/or different developmental stages, such as egg secretome studies which have led to the discovery of highly sensitive antigens for the diagnosis of schistosomiasis (131–135) may facilitate the identification of additional molecules with therapeutic potential. An increasing number of candidates applicable for therapy purpose have been identified from other helminths (136), specific examples being anti-inflammatory protein-2 (AIP-2) from hookworm (137) and EgKI-1, a potent Kunitz type protease inhibitor from *Echinococcus granulosus* (126); on this basis, one could speculate that the schistosome orthologs of these molecules would likely elicit similar therapeutic effects. In addition, if these and other orthologs can provide the requisite therapeutic efficacy, in-depth analysis of the underlying molecular regulatory mechanisms would follow.

(4) Further work should be undertaken on schistosome extracellular vesicles (EVs), which as indicated earlier, are small membrane-bounded secreted vesicles that can transmit a wealth of bioactive cargos, such as proteins, lipids, glycans, DNA, messenger RNAs (mRNAs) and miRNAs between cells thereby playing a key role in cell-cell communication (138). A number of EV components have already been identified in schistosomes (18, 139–141), providing a basis for their application as biomarkers for human schistosomiasis, as novel vaccine targets (142, 143) or as modulators of the host immune response. Indeed, it has been recently shown that *S. japonicum* EVs can be taken up primarily by macrophages and other host immune cells when the miRNA cargo (miR-125b and bantam) is transferred to recipient cells; this promotes macrophage proliferation and TNF-α production by regulating targets including *Pros1*, *Fam212b*, and *Clmp* and emphasizes the ability of *Schistosoma* EV components to modulate the host immune response thereby helping to facilitate parasite survival (144). Furthermore, *S. mansoni* EV‐enclosed miRNAs have been shown to modulate host T helper cell differentiation (145). It will be intriguing to determine whether this immune-regulatory propensity of schistosomes can be harnessed for the future treatment of human autoimmune and inflammatory diseases and cancer.

(5) As of Dec 11, 2020, the COVID-19 pandemic had caused 1,586,047 deaths around the world and accumulating evidence showed that there is a higher concentration of pro-inflammatory cytokines, such as IL-6, in severe cases compared with moderate cases (146). An increasing number of studies indicate that the “cytokine storm” may contribute to the mortality of COVID-19, most likely induced by the IL-6 amplifier (147). A couple of Disease-modifying anti-rheumatic drugs, such as tocilizumab and hydroxychloroquine, have been proposed as potential immune-modulating therapies for the treatment of COVID-19 (148). Many of the *Schistosoma* components listed in **Table 1** are prone to induce Th2 and Treg immune responses; it would be informative to evaluate whether some of these components, such as rSjCystatin and SJMHE1, could be potential preventive and/or therapeutic drug candidates for severe COVID-19. On the other hand, some of the *Schistosoma* molecules shown to suppress immunological disorders may impair the protective efficacy, in schistosomiasis-endemic populations, of antibacterial and antiviral vaccines, notably those in development against SARS-CoV-2 which tend to induce a Th1-biased immune response (149). Similarly, such schistosome components may have a potential detrimental effect on cancer treatments, which usually require a Th1 immune response for effectiveness (150). The future clinical application of *Schistosoma* components should, therefore, be carefully scrutinized before deployment at scale.

## Conclusions

As mass deworming programs are increasingly implemented in developing countries, the incidence of autoimmune and inflammatory diseases can be expected to rise in the forthcoming decades considering the basic concept of the “Old Friends” hypothesis. Controlled helminth infections and/or helminth-derived products may become new weapons for the prevention and/or cure of these disorders. Although there are still considerable challenges, particularly in regard to undertaking safe human clinical trials, schistosome-derived products may play an important future role as immunotherapies for acute and chronic inflammatory diseases, particularly when the underpinning immunomodulatory mechanisms are further explored and revealed.

## Author Contributions

This manuscript was conceptualized by PC, drafted by YM and NH, and revised by DM and PC. All authors contributed to the article and approved the submitted version.

## Funding

This work was funded by the National Health and Medical Research Council (NHMRC) of Australia (ID: APP1160046, APP1102926, APP1037304, and APP1098244). DPM is a NHMRC Senior Principal Research Fellow and Senior Scientist at QIMRB. The funders had no role in study design, data collection and analysis, decision to publish, or preparation of the manuscript.

## Conflict of Interest

The authors declare that the research was conducted in the absence of any commercial or financial relationships that could be construed as a potential conflict of interest.
